# Ultrasound-guided transversus abdominis plane block in obese cats: a preliminary cadaveric study

**DOI:** 10.1177/1098612X251329326

**Published:** 2025-04-29

**Authors:** Marta Garbin, Beatriz P Monteiro, Paulo V Steagall

**Affiliations:** 1 Faculty of Veterinary Medicine, Université de Montréal, Department of Clinical Sciences, Saint-Hyacinthe, QC, Canada; 2 Jockey Club College of Veterinary Medicine and Life Sciences, City University of Hong Kong, Department of Veterinary Clinical Sciences, Hong Kong SAR, China; 3 Centre for Animal Health and Welfare, City University of Hong Kong, Hong Kong SAR, China

**Keywords:** Analgesia, local anesthetic, locoregional anesthesia, lean body weight, actual body weight, transversus abdominis plane block, pain

## Abstract

**Objectives:**

The aim of the present study was to investigate the distribution of adipose tissue in the abdominal wall of obese cats and compare the injectate spread and spinal nerve staining after ultrasound-guided transversus abdominis plane (TAP) block using lean (LBW) vs actual body weight (ABW).

**Methods:**

Four cat cadavers with a body condition score ⩾8/9 were included. Cat 1 was dissected to identify the TAP and describe abdominal fat distribution. Cats 2 and 3 received a two-point ultrasound-guided TAP injection of 0.25 ml/kg/point based on LBW and ABW, respectively. In cat 4, both hemiabdomens were randomly injected with the two volumes. Subsequent anatomic dissection assessed injectate distribution and the number of thoracic (T) and lumbar (L) spinal nerves stained ⩾1 cm circumferentially.

**Results:**

The mean weight of the cats was 7.5 ± 0.3 kg and they had a body condition score of 9/9. A thick layer of hypoechoic adipose tissue was observed ventral to the costal arch, between the rectus and transversus abdominis muscles, and a second thinner layer between the obliquus internus and transversus abdominis muscles. After crossing the adipose tissue, the ventral branches of spinal nerves lie in the fascial plane, superficial to the transversus abdominis muscle. LBW- and ABW-based injectate volumes stained the ventral branches from T12 to L1 and T11 to L1, respectively.

**Conclusions and relevance:**

Two separate layers of adipose tissues are localized superficially to the transversus abdominis muscle in obese cats. Identifying the transversus abdominis muscle and adipose layers is crucial for the success of the TAP block. Injectate volumes based on ABW may provide wider staining of thoracolumbar spinal nerves than LBW. Further randomized clinical trials are needed in obese cats using locoregional anesthesia.

## Introduction

Obese cats, similar to obese humans,^1,2^ may benefit from locoregional analgesic techniques to control perioperative pain while reducing the administration of opioids and their related adverse effects. The transversus abdominis plane (TAP) block is a fascial plane block, which consists of injecting a local anesthetic into the TAP where the thoracolumbar nerves that supply the abdominal wall are located, inducing desensitization of the abdominal wall and parietal peritoneum of the cranial and middle abdominal regions.^3^ Ultrasound guidance is employed to identify the TAP and increase the technique’s success rate and safety.^4^ However, in obese patients, the thickness and distribution of the adipose tissue make it difficult to visualize the anatomic landmarks of interest.^5^

An ultrasound-guided TAP block with local anesthetics may be used for the management of abdominal wall pain in dogs^6–11^ and cats.^12–14^ On the other hand, the most efficient technique and volume of injectate have yet to be determined. A single volume of 0.5 ml/kg/hemiabdomen has been investigated in anatomic^15,16^ and clinical studies^14^ in cats of normal weight. However, knowing that fascial plane blocks rely on a high volume of local anesthetics, it is possible that a larger volume of injectate would be necessary for the TAP block in obese cats as this population may have a larger abdominal surface area than non-obese cats.

This anatomic preliminary study describes the distribution of adipose tissue in the abdominal wall of obese cats and spinal nerve staining after a two-point TAP block based on lean (LBW) vs actual body weight (ABW). We hypothesized that the ABW-based volume of injectate would stain a wider number of thoracolumbar spinal nerves within the TAP than the LBW-based volume.

## Materials and methods

This randomized, blinded, cadaveric descriptive study was approved by the Comité d’éthique de l’utilisation des animaux of the Université de Montréal (21-Rech-2111).

Four frozen cadavers of adult domestic shorthair cats with a body condition score (BCS) ⩾8 on a scale^17^ of 1–9 and no evidence of skeletal and/or muscular injuries nor signs of previous abdominal surgery were included. The cadavers were previously owned cats euthanized for reasons unrelated to the study and donated to the Centre Hospitalier Universitaire Vétérinaire of the Université de Montréal for teaching and research purposes.

Cat 1 was used to study the gross anatomy of the abdominal wall, with attention to the distribution of adipose tissue. One hemiabdomen of cats 2 and 3 was used to study the sonoanatomy of the TAP and surrounding tissues. The contralateral hemiabdomens of cats 2 and 3, as well as both hemiabdomens of cat 4, were used to assess the injectate spread and nerve staining after TAP injections by dissection.

Each cadaver was thawed at room temperature for 48 h before the experiment. Then, the thoracic and abdominal areas were clipped. The LBW-based TAP injection was performed first, followed by the ABW-based injection. One investigator (MG) randomly selected the hemiabdomen allocation using a randomization order by Microsoft Excel (Microsoft Corporation). A second investigator (BPM) assessed the BCS^17^ and estimated the LBW of each cat based on clinical experience, considering that each BCS point above the appropriate weight indicates a 7–10% increase in body weight.^18^

All injections were performed by the same anesthesiologist (MG) using a 22 G, 50 mm Quincke spinal needle (BD spinal needle; Becton Dickinson & Co) connected by a T-port (Med-RX extension set with t-connector; CHS) to a prefilled 5 or 10 ml syringe, with the cat in dorsal recumbency. A 38 mm, 13–6 MHz linear transducer connected to a portable ultrasound machine (Edge II; Sonosite) was used to visualize the target plane and perform the injections. The ultrasound was initially set at the minimum depth of 1.9 cm and the image was optimized by adjusting the gain.

**Figure 1:**
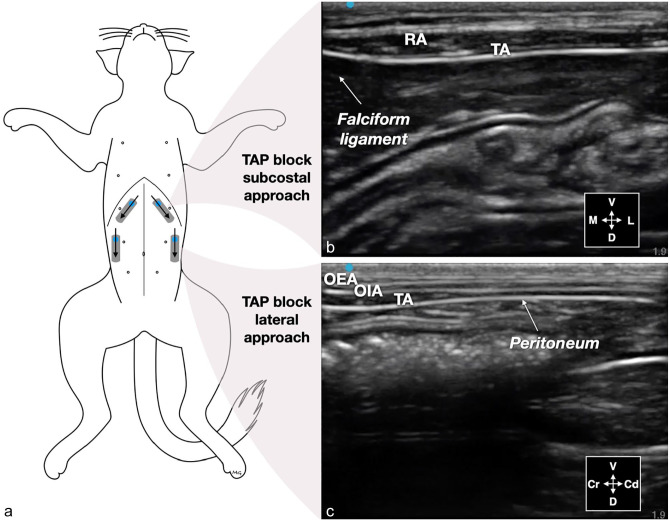
Ultrasound-guided transversus abdominis plane (TAP) block in a normal-weight cat: (a) schematic representation of the transducer position (gray rectangles) and needle orientation (black arrows) to perform a bilateral two-point (subcostal and lateral–longitudinal) TAP block; (b) ultrasound image of the TAP block subcostal approach before administration of injectate solution between the rectus abdominis (RA) and transversus abdominis (TA) muscles; and (c) ultrasound image of the TAP block lateral–longitudinal approach before the administration of injectate between the obliquus internus abdominis muscle and the TA. Cd = caudal; Cr = cranial; D = dorsal; L = lateral; M = medial; OEA = obliquus externus abdominis muscle; V = ventral. Adapted from Garbin et al^14^

In the two hemiabdomens used to study the sonoanatomy, 0.2 ml of a bupivacaine-dye solution was injected into the TAP. Then, a dissection was performed to verify the injection was in the correct plane. In the four hemiabdomens used to study the injectate distribution, a volume of 0.25 ml/kg/point based on LBW or ABW of the bupivacaine-dye solution was injected using a two-point (subcostal/lateral-longitudinal) ultrasound-guided TAP injection as previously described^16^ (Figure 1). The injectate solution was prepared by diluting 0.2 ml of permanent tissue dye (Davison Marking System; Bradley Products) with 20 ml of bupivacaine hydrochloride 0.25% (Bupivacaine Injection BP 0.25%; SteriMax).

Approximately 45 mins after injection, cadavers were dissected by two investigators (BPM and MG). With the cat in dorsal recumbency, skin and subcutaneous adipose tissue were incised along the ventral midline from the thorax to the pubis and reflected laterally. After identification of the rectus and obliquus externus abdominis muscles, the aponeurosis of the latter was dissected longitudinally along the rectus abdominis and the belly of the muscle was reflected laterally. The rectus abdominis sheath was dissected from its lateral margin and the muscle reflected medially. The terminal ending of the thoracolumbar spinal nerves was identified and followed dorsally. Then, the aponeurosis of the muscle obliquus internus abdominis was dissected and reflected laterally to expose the spinal nerve branches superficial to the transversus abdominis muscle. The ventromedial branches of the spinal nerves from the tenth thoracic (T) to the second lumbar (L) were considered the target nerves. Nerves were assessed as successfully stained when their entire circumference was dyed for a length ⩾1 cm.^15,16^ After nerve staining evaluation by an investigator unaware of injectate volume (BPM), an incision was made along the linea alba to expose the abdominal cavity and examine the abdominal organs for dye staining. Any presence of dye in the muscles, adipose tissue, non-target area or abdominal cavity was noted. The time taken to perform the TAP block was recorded.

The Shapiro–Wilk test was used to assess data distribution (SPSS Statistics; IBM). Normally and non-normally distributed data are presented as mean ± SD and median (range), respectively. Descriptive statistics were used to report the injectate distribution and nerve staining. The success rate in nerve staining was reported as the number of nerves stained with the two volumes divided by the number of target nerves per hemiabdomen (n/N) and as a percentage.

## Results

Two castrated male and two spayed female cats with a mean body weight of 7.5 ± 0.3 kg and BCS of 9/9 were used. LBWs of 5.2, 5.0 and 5.4 kg were assigned to cats 2, 3 and 4, respectively.

**Figure 2:**
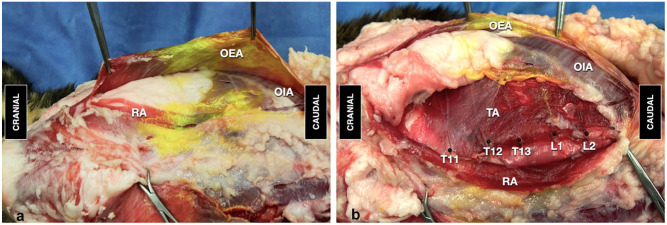
View of the abdominal wall after skin and subcutaneous tissue removal showing the distribution of dye administered by an ultrasound-guided two-point transversus abdominis plane (TAP) injection in an obese cat cadaver. (a) Adipose tissue localized in the cranial region of the abdomen deeper to the rectus abdominis (RA) and obliquus externus abdominis (OEA), and on the caudal flank between the obliquus internus abdominis (OIA) and transversus abdominis (TA) muscles. (b) Ventromedial branches of thoracic (T) and lumbar (L) nerves localized in the TAP (black pins)

Gross anatomic dissection revealed a layer of subcutaneous adipose tissue and a broad layer of fat in the cranial portion of the abdomen, caudal and ventral to the last rib. This thick layer extended caudally between the rectus abdominis and transversus abdominis in the cranioventral region of the abdomen and between the obliquus externus and internus abdominis muscles in the subcostal–lateral region of the abdomen. A second thin layer separated the obliquus internus and transversus abdominis muscles in the laterocaudal region of the abdomen. Adipose tissue infiltrated the aponeurosis of the obliquus internus abdominis (Figure 2).

**Figure 3:**
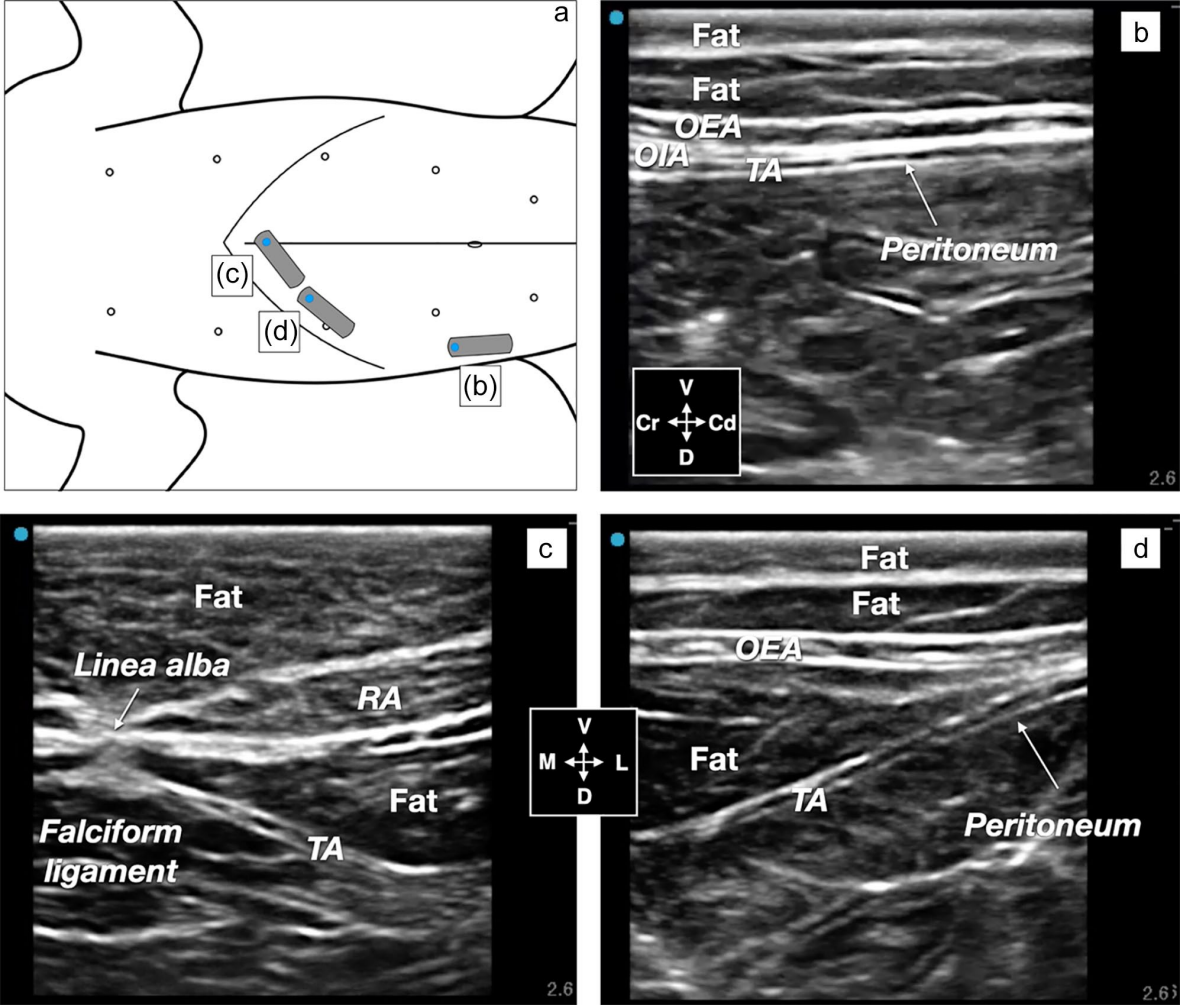
Ultrasonographic identification of the transversus abdominis plane, abdominal muscles and adipose tissue in a cat cadaver with a body condition score of 9/9. (a) Schematic representation of the transducer positions in the cat in the dorsal position. The gray rectangles indicate the positions of the transducer, and the blue dot is the transducer marker (orientation). (b–d) Ultrasound images of the abdominal wall corresponding to the transducer in positions: (b), lateral–longitudinal to the mammary line and the level of the umbilicis; (c), close to the linea alba; and (d), subcostal at the level of the mammary line. Cd = caudal; Cr = cranial; D = dorsal; L = lateral; M = medial; OEA = obliquus externus abdominis muscle; OIA = obliquus internus abdominis muscle; RA = recus abdominis muscle; TA = transversus abdominis muscle; V = ventral

Ultrasonographic visualization of the abdominal wall is shown in Figure 3. At the level of the subcostal approach, the adipose tissue was observed as hypoechoic layers between the transversus abdominis and rectus abdominis muscles ventrally, and the obliquus externus and transversus abdominis laterally. The echogenicity between the adipose tissue and abdominal muscles was difficult to distinguish. Several longitudinal hyperechoic lines were observed within and around the fat layers. The hyperechoic peritoneum and the hypoechoic transversus abdominis muscle were used as ultrasonographic landmarks for TAP identification.

Ultrasonographic visualization of injectate distribution within the TAP was successfully achieved with all injections. The mean time to perform the block was 289 ± 4 s. Anatomic dissection revealed that all injections were performed in the TAP. The target branches of the spinal nerve from T10 to L2 were identified within the TAP in all cadavers. The branches from T12 to L1 and from T11 to L1 were stained with LBW- and ABW-based injectate volumes, respectively (Table 1). The overall success rate was 50% with the LBW-based volume and 67% with the ABW-based volume. Dye was also observed on the dorsal margin of the adipose layer between the rectus and transversus abdominis muscles and in the fascial plane between the aponeuroses of obliquus internus and externus abdominis (Figure 2). No dye was found in the abdominal cavity.

**Table 1:** Staining of the ventral branches of the thoracic (T) and lumbar (L) spinal nerves into the transversus abdominis plane using a colored injectate volume based on lean body weight (LBW) and actual body weight (ABW)

	Volume based on LBW							Volume based on ABW						
	T10	T11	T12	T13	L1	L2	Success rate	T10	T11	T12	T13	L1	L2	Success rate
Cat 2	No	No*	Yes	Yes	Yes	No	3/6 (50)	–	–	–	–	–	–	–
Cat 3	–	–	–	–	–	–	–	No	Yes	Yes	Yes	Yes	No	4/6 (67)
Cat 4	No	No*	Yes	Yes	Yes	No	3/6 (50)	No*	Yes	Yes	Yes	Yes	No*	4/6 (67)

Values are given as n (%). Successful staining was considered when the dye was found along the circumference of the nerve for at least 1 cm in length (Yes)

* Staining of the nerve <1 cm length circumferentially or in less than the full circumference

## Discussion

The TAP block has been used successfully in the context of multimodal analgesia for abdominal surgeries in normal-weight cats.^14^ However, evidence in obese patients is still lacking. There is an interest in administering the TAP block in obese cats, as these patients may be at increased risk of postoperative pulmonary complications or concomitant comorbidities.^1,2,5,18,19^ Ultrasound guidance increases the success rate of the TAP block.^4^ However, identifying the TAP can be challenging,^20^ requires training and may increase anesthesia/surgery time.^21^

This preliminary study described the anatomic and ultrasonographic findings of the TAP in obese cats, including the distribution of local anesthetic and nerve staining. Ultrasound-guided TAP injections were successfully performed in three obese cats; however, approximately 5 mins were required to perform the technique per each hemiabdomen. Therefore, the same operator took twice as long to perform the TAP in obese cats than in non-obese cats.^14^ Identification of the transversus abdominis muscle at the level of the subcostal injection point was more challenging than its lateral injection point because of several layers of fat and ultrasound settings that are used in normal-weight cats.^15,16^ By increasing the scanning depth, it was finally possible to recognize the falciform ligaments and the peritoneum and presume that the hypoechoic muscle superficial to the falciform ligaments was the transversus abdominis muscle. A second confirmation was achieved by sliding the ultrasound transducer laterally along the last rib and following the transversus abdominis muscle until the thick superficial fat layer disappeared (Figure 3). At the lateral injection point, the hyperechoic peritoneum and the real-time movement of the abdominal organs facilitated the identification of the transversus abdominis muscle. Therefore, setting the ultrasound image depth at 2.6 cm (or more) is recommended to identify the relevant anatomy and transversus abdominis muscle in obese cats. The image depth should then be gradually reduced to facilitate accurate needle-tip visualization in the target plane.

Fascial plane blocks require high-volume local anesthetics to spread within the fascial plane and desensitize spinal nerves. However, only a conventional volume of 1 ml/kg/cat has been investigated in normal-weight cats.^15,16^ In this preliminary study, the ABW-based volume was approximately 40–45% more than the volume calculated on LBW, leading to wider spread and higher successful staining of target nerves. In obese patients, recommended doses of anesthetics are usually calculated using LBW to reduce the risks of toxicosis.^22^ Therefore, although this study indicates that anesthetic volume based on ABW may be beneficial, maximum doses of local anesthetics should be first calculated using LBW. Larger volumes of injections could then be achieved by adding saline up to the intended volume of administration.

## Conclusions

In obese cats, ultrasound identification of the abdominal wall muscles is challenging owing to excessive adipose tissue. In this study, fat was distributed subcutaneously in the abdominal wall, between the rectus abdominis and transversus abdominis muscles in the cranioventral region of the abdomen and within the TAP in the caudolateral region. Clinically, a TAP block with a volume based on ABW may desensitize more thoracolumbar spinal nerves than LBW. However, the anesthetic dose should be based on LBW. Future studies involving large sample sizes in the clinical setting are warranted.
